# Innate Immune Deficiency of Extremely Premature Neonates Can Be Reversed by Interferon-γ

**DOI:** 10.1371/journal.pone.0032863

**Published:** 2012-03-12

**Authors:** Pierre Tissières, Agnieszka Ochoda, Irène Dunn-Siegrist, Geneviève Drifte, Michel Morales, Riccardo Pfister, Michel Berner, Jérôme Pugin

**Affiliations:** 1 Division of Intensive Care, University Hospitals of Geneva, Geneva, Switzerland; 2 Division of Neonatal and Pediatric Intensive Care, University Hospitals of Geneva, Geneva, Switzerland; 3 Division of Obstetrics, University Hospitals of Geneva, Geneva, Switzerland; 4 Department of Microbiology and Molecular Medicine, Faculty of Medicine, University of Geneva, Geneva, Switzerland; Louisiana State University, United States of America

## Abstract

**Background:**

Bacterial sepsis is a major threat in neonates born prematurely, and is associated with elevated morbidity and mortality. Little is known on the innate immune response to bacteria among extremely premature infants.

**Methodology/Principal Findings:**

We compared innate immune functions to bacteria commonly causing sepsis in 21 infants of less than 28 wks of gestational age, 24 infants born between 28 and 32 wks of gestational age, 25 term newborns and 20 healthy adults. Levels of surface expression of innate immune receptors (CD14, TLR2, TLR4, and MD-2) for Gram-positive and Gram-negative bacteria were measured in cord blood leukocytes at the time of birth. The cytokine response to bacteria of those leukocytes as well as plasma-dependent opsonophagocytosis of bacteria by target leukocytes was also measured in the presence or absence of interferon-γ. Leukocytes from extremely premature infants expressed very low levels of receptors important for bacterial recognition. Leukocyte inflammatory responses to bacteria and opsonophagocytic activity of plasma from premature infants were also severely impaired compared to term newborns or adults. These innate immune defects could be corrected when blood from premature infants was incubated *ex vivo* 12 hrs with interferon-γ.

**Conclusion/Significance:**

Premature infants display markedly impaired innate immune functions, which likely account for their propensity to develop bacterial sepsis during the neonatal period. The fetal innate immune response progressively matures in the last three months *in utero*. *Ex vivo* treatment of leukocytes from premature neonates with interferon-γ reversed their innate immune responses deficiency to bacteria. These data represent a promising proof-of-concept to treat premature newborns at the time of delivery with pharmacological agents aimed at maturing innate immune responses in order to prevent neonatal sepsis.

## Introduction

Twenty percent of premature infants surviving beyond the first three days of life will develop one or more culture-proven bacteraemic sepsis. As much as 46% of infants born before 25 weeks of gestational age will develop sepsis [1]. Coagulase-negative staphylococci, mainly *S.epidermidis*, are responsible for half of all sepsis, and the rest is caused by other Gram-positive bacteria and Gram-negative bacteria such as *E.coli* [1], [2]. The prevalence of neonatal sepsis is inversely correlated with gestational age and birth weight, and is associated with morbid complications such as bronchopulmonary dysplasia, cerebral palsy, an increased hospital length of stay, and a high mortality rate [1], [3]. Whereas sepsis secondary to coagulase-negative staphylococci, is rarely fatal, neonatal sepsis due to Gram-negative bacteria is associated with an elevated mortality [2].

The innate immune system is essential for the newborn's defence against microorganisms during the first months of life. Adaptive immunity is still immature, and immunological memory only starts to develop. In neonates, innate immune defences against bacteria rely on soluble proteins (mainly opsonins, complement and maternal immunoglobulins) and phagocytes such as neutrophils. Hypogammaglobulinemia and low complement activity have been reported in premature infants and may play a role in their impaired immunity against bacteria. When measured, levels of opsonins such as collectins were found to be low in premature infants [4], [5].

The recognition of the presence of bacteria as nonself and dangerous intruders is complex and involves an array of receptors present at the surface of host leukocytes, mainly phagocytes. The main receptors involved in this process are Toll-like receptors (TLRs) and associated molecules. TLRs recognize and bind conserved microbial molecules, such as lipopolysaccharide (LPS) from Gram negative bacteria (TLR4/MD-2 complex) and lipopeptides from Gram-positive and Gram-negative bacteria (TLR2) [6]. CD14 is a pattern recognition receptor binding and transferring various bacterial molecules to TLRs [7]. TLRs start to be expressed in foetal tissues as early as during the 18–21 weeks of gestational age [8]. Monocyte TLR4 and CD14 expression levels were found to be lower in premature neonates as compared to term newborns and adults [9], [10], [11]. The TLR4-associated molecule MD-2, essential for LPS recognition and binding, was found in term infants and adult Paneth cells but not in premature neonates [12]. We hypothesized that the increased prevalence of neonatal sepsis in extremely premature infants was associated with an immaturity of their innate immune system. We measured the expression of receptors of the innate immunity in leukocytes from cord blood in premature neonates, tested their response to bacteria and the capacity of their plasma to enhance bacterial phagocytosis. We finally tested if a short *ex vivo* treatment with interferon-γ could boost innate immune functions of premature neonate leukocytes.

## Results

### Characteristics of patients

Ninety subjects were included. Characteristics of the 70 infants (21 extremely low birth weight, ELBW, infants of less than 28 wks of gestational age; 24 very low birth weight, VLBW, infants born between 28 and 32 wks of gestational age; and 25 term newborn) are shown in Table 1. Gestational age, birth weight, Apgar score at 5 minutes, and frequency of caesarean delivery were statistically different between infant groups, whereas mother age, maternal diabetes, prolonged rupture of the membranes were not statistically different. Although not different between ELBW and VLBW infants, maternal pre-eclampsia was statistically more frequent in premature than in term newborn. White blood cell count was not statistically different between ELBW and VLBW infants, but was lower in both groups when compared with term newborns (*P<0.001*). The adult control group (n = 20) median age was 32 yrs (interquartile range, IQR: 28.7–36.2 yr.), and male∶female ratio was 12∶8.

**Table 1:** Baseline Characteristics and outcome of newborns.

	ELBW	VLBW	Term Newborn
	N = 21	N = 24	N = 25
Gestational age – weeks	26 [24]–[27]	31 [29]–[32]	39 [38]–[41]
Weight – grams	705 [600–850]	1495 [1135–1763]	3310 [2970–3650]
Male gender no. (%)	9 (42.8)	12 (50)	13 (52)
Apgar score at 5 min	7 [2]–[8]	8 [7]–[9]	10 [10–10]
WBC count at delivery (G/L)	9.2 [6.0–12.7]	9.8 [6.6–12.8]	17.5 [15.7–25.9] †
Neutrophils count (G/L)	5.3 [2.1–6.2]	3.7 [2.5–5.9]	11.4 [9.3–16.5] †
Lymphocytes count (G/L)	3.3 [2.3–4.2]	4.5 [2.4–5.9]	4.6 [3.3–6.4] †
Monocytes count (G/L)	0.7[0.4–1.1 ]	0.8 [0.5–1.2]	2.4 [1.5–5.7] †
Cesarean delivery no.(%)	9 (42.8)	18 (75)	2 (8)
Duration of Hospital Stay – days	1 [1–58]	30 [8]–[47]	2[1]–[3]
Mother age – years	33 [27]–[35]	32 [30]–[34]	31 [28–35.2]
Maternal pre-eclampsia	5 (23.8)	7 (21.2)	0 (0)
Maternal diabetes	2 (9.5)	0 (0)	0 (0)
Rupture of the membranes for >24 hrs	2 (9.5)	4 (16.6)	6 (24)
Late onset sepsis	4 (19)	1 (4.1)	0 (0)
Early onset sepsis	0 (0)	1 (4.1)	0 (0)
IVH grade 4	3 (14.3)	1 (4.1)	0 (0)
Death	14 (66.7)	2 (8.3)	0 (0)

Continuous data are expressed as median and interquartile range [IQR] and categorical data as number (%).

ELBW, extremely low birth weight born before 28 wks of gestational age; VLBW, very low birth weight born between 28 and 32 wks of gestational age, WBC, white blood cell; IVH, intraventricular haemorrhage. †, WBC count was performed in term newborn if there was an infectious risk at delivery (WBC count performed in ELBW, N = 13; VLBW, N = 24; and in term newborns, N = 8). Four premature infants (ELBW, N = 3; VLBW, N = 1) did not receive pulmonary maturation with antenatal corticosteroid therapy.

### Premature infants have a low leukocyte surface expression of TLR2, TLR4, CD14 AND MD-2

**Figure 1:**
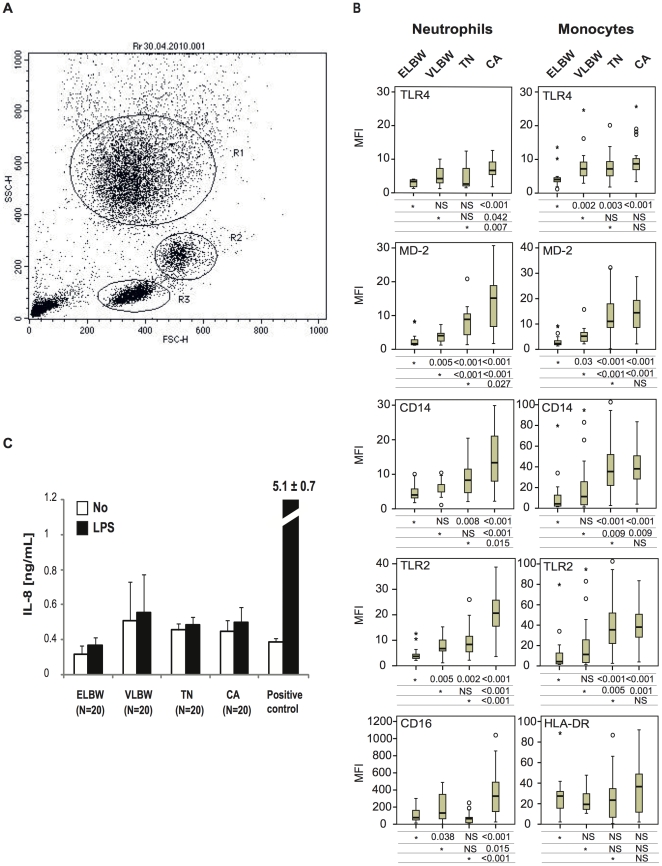
TLR2, TLR4, CD14, and MD-2 surface expression of blood leukocytes and plasma soluble MD-2 activity from premature infants, term newborns, and control adults. (A) Representative flow cytometry plot showing forward and side-scatter characteristics gating used to identify neutrophils (R1), and monocytes (R2). (B) Surface expression of TLR4, MD-2, CD14 and TLR2 of phagocytes (neutrophils and monocytes) from extremely low birth weight infants (ELBW, born before 28 wks of gestational age, N = 18), very low birth weight infants (VLBW, born between 28–32 wks of gestational age, N = 17), term newborn (TN, N = 25), and control adult (CA, N = 20). In addition the expression of the Fcγ receptor CD16 on neutrophils and the major histocompatibility HLA-DR on monocytes are shown. Receptor expression was measured by flow cytometry, and expressed as mean fluorescence index (MFI, geo mean receptor/geo mean IgG control). Errors bars are means ± SEM, On each line, a reference group (*) is compared to other groups and respective P value expressed using Mann-Whitney *U* test. (C) Plasma soluble MD-2 activity was measured as the capacity of plasma to support TLR4-HEK293 cell activation after a 30 ng/mL LPS challenge [50]. Human recombinant soluble MD-2 (1 µg/mL) was used as a positive control. (ELBW, extremely low birth weight premature infants born before 28 wks of gestational age N = 20; VLBW, very low birth weight premature infants born between 28–32 wks of gestational age, N = 20; TN, term newborns, N = 20; CA, control adults, N = 20). Errors bars are means ± SEM.

Receptor surface expression was assessed in 80 subjects after gating subpopulations of circulating leukocytes (Figure 1A, and 1B). Neutrophils from ELBW newborns had a significantly reduced TLR2, TLR4, CD14 and MD-2 surface expression when compared to adults. Whereas surface expression of MD-2, CD14 and TLR2 significantly increased with gestational age, TLR4 expression was found to be similar in premature and term newborns. Only TLR2, TLR4, and CD14, but not MD-2 expression, was found to be lower in term newborn neutrophils as compared with control adults. CD16 surface expression was lower in neutrophils from all infants compared to adults. Monocyte HLA-DR expression was not statistically different between groups. Monocytes TLR4 expression was found to be lower only in ELBW infants. Monocyte TLR4 expression levels were not statistically different between VLBW, term newborns and control adults. Monocyte expression of MD-2, CD14 and TLR2 were lower in both ELBW and VLBW infants as compared to term newborns and adults. No significant difference in monocytes TLR2, TLR4 and MD-2 surface expression was found between term newborns and adults (Figure 1B).

We next investigated whether a low surface MD-2 expression observed in premature phagocytes could be balanced by an increased level of the soluble form of MD-2 (sMD-2) in plasma from 80 subjects. LPS/sMD-2-dependent activation of TLR4-expressing cells was found to be similar in all groups tested (Figure 1C), suggesting that - at basal level - no significant sMD-2 activity was detected in premature infants, term newborns, and adults.

### Defective opsonophagocytosis in premature infants

**Figure 2:**
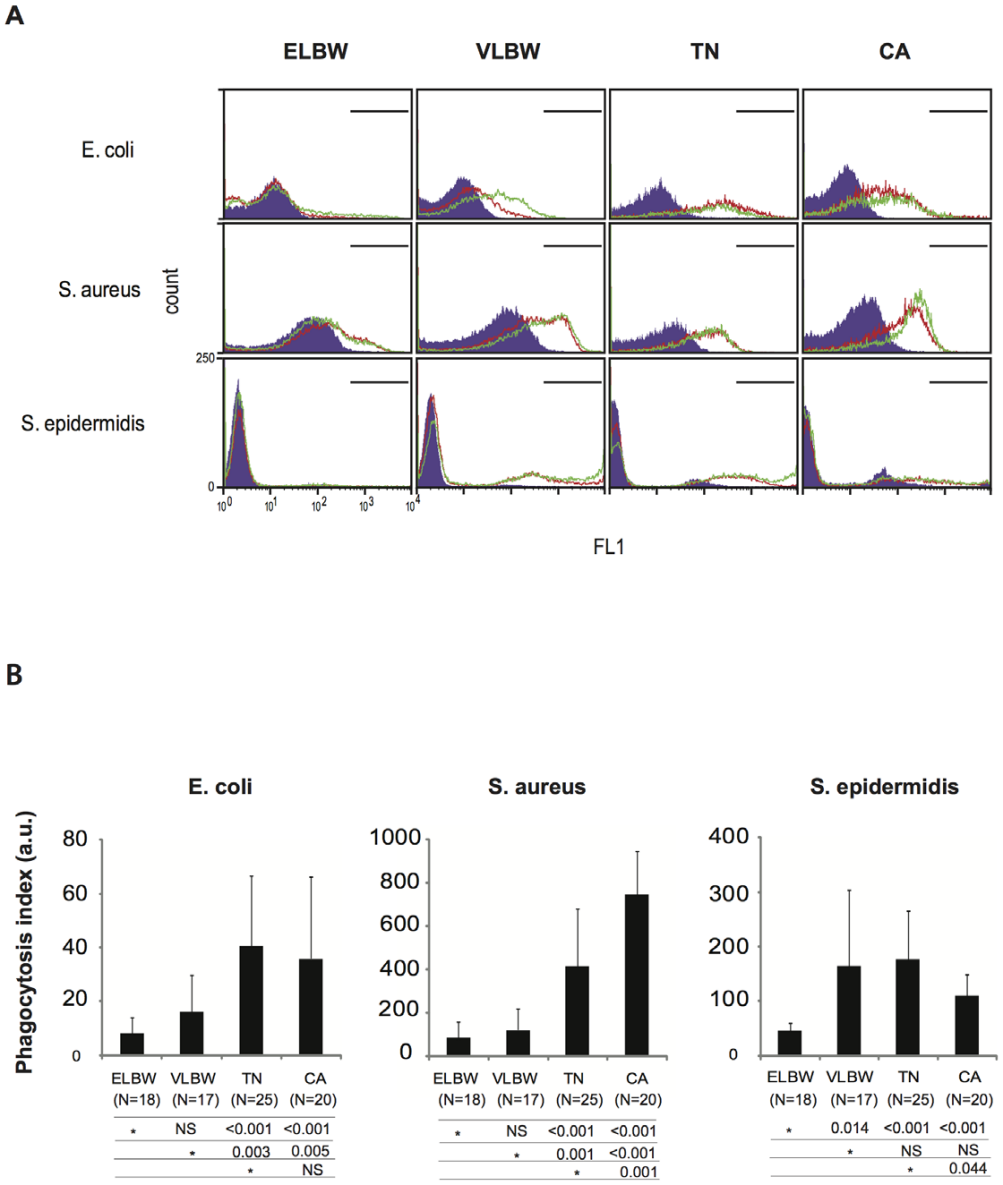
Phagocytosis of bacteria by newborn and adult neutrophils, and plasma opsonic activity. (A) Phagocytosis of fluorescent *E.coli*, *S.aureus*, and *S.epidermidis* by neutrophils from one representative subject from each group (ELBW, extremely low birth weight premature infants born before 28 wks of gestational age; VLBW, very low birth weight premature infants born between 28–32 wks of gestational age; TN, term newborns; and CA, control adults). Phagocytosis was measured by flow cytometry after 10 min (red lines) and 20 min (green lines) incubation with bacteria opsonised with autologous plasma. Neutrophils pre-incubated with the phagocytosis blocker cytochalasin D are presented with filled curves (negative control). (B) The opsonic capacity of patient's plasma using neutrophil-like human HL-60 cells is tested. Fluorescent *E.coli*, *S.aureus*, and *S.epidermidis* were opsonised with autologous plasma and incubated 40 min with DMSO-differentiated HL-60 cells. Intracellular fluorescence (phagocytosis of bacteria) was measured by flow cytometry and expressed as mean phagocytic index (± SEM) of the four groups (ELBW, extremely low birth weight premature infants born before 28 wks of gestational age N = 21; VLBW, very low birth weight premature infants born between 28–32 wks of gestational age, N = 24; TN, term newborns, N = 25; CA, control adults, N = 20). On each line, a reference group (*) is compared to other groups and respective P value expressed using Mann-Whitney *U* test.

We used the well-behaved and consistent DMSO-differentiated HL-60 cells as a surrogate neutrophil-like target cell to measure patient plasma-dependent opsonophagocytosis of bacteria (Figure S1). Plasma from premature infants carried a significantly lower opsonophagocytic capacity than term newborns and adults for *E.coli* and *S.aureus* (Figure 2A, B). Plasma from ELBW infants had a lower opsonophagocytic capacity to *S.epidermidis* compared to VLBW infants, term newborns and adults (Figure 2A,B). The opsonic capacity of adult plasma to *S.aureus* was markedly higher than that of newborns, suggesting the acquisition of *S.aureus* opsonins during the post-natal period. In a subset of patients, opsonophagocytosis was tested with primary neutrophils as target phagocytes, and confirmed the defective phagocytic capacity to *E.coli*, *S.aureus*, and *S.epidermidis* in ELBW observed using HL-60 cells (Figure S2).

### Defective inflammatory response to bacteria in leukocytes from premature infants

**Figure 3:**
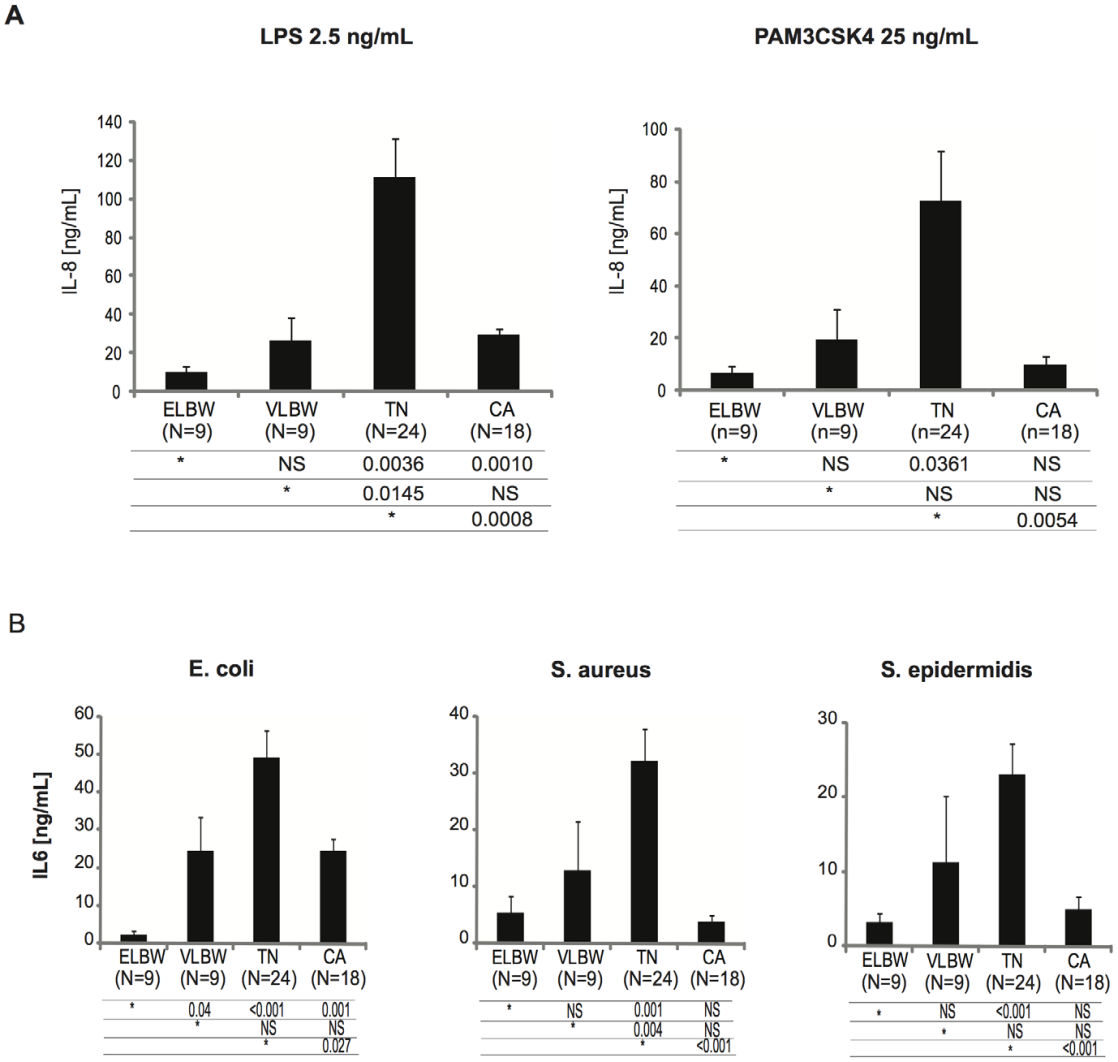
Whole blood response to bacterial agonists and heat-killed bacteria. (A) Whole blood obtained from subjects of the four groups (ELBW, extremely low birth weight premature infants born before 28 wks of gestational age, N = 9; VLBW, very low birth weight premature infants born between 28–32 wks of gestational age, N = 9; TN, term newborns, N = 18; CA, control adults, N = 24) were stimulated 12 hrs with LPS (2.5 ng/mL) or PAM_3_CSK_4_ (25 ng/mL). Interleukin-8 levels (mean ± SEM) were quantified by ELISA in conditioned plasma. On each line, a reference group (*) is compared to other groups and respective P value expressed using Mann-Whitney *U* test. (B) Whole blood obtained from subjects of the four groups (ELBW, extremely low birth weight premature infants born before 28 wks of gestational age, N = 9; VLBW, very low birth weight premature infants born between 28–32 wks of gestational age, N = 9; TN, term newborns, N = 18; CA, control adults, N = 24) were stimulated 12 hrs with heat-killed bacteria (3.7×10^6^ *E. coli*, *S. epidermidis*, and 1.56×10^6^ *S. aureus*). Interleukin-6 levels (mean ± SEM) were quantified by ELISA in conditioned plasma. On each line, a reference group (*) is compared to other groups and respective P value expressed using Mann-Whitney *U* test.

Premature infants, particularly ELBW infants, had a lower inflammatory response to LPS, and to a lower extent to the lipopeptide PAM_3_CSK_4_ (Figure 3A). Interestingly, term newborns showed a significantly higher inflammatory response than control adults. Similarly to purified bacterial agonists, the inflammatory response to whole heat-killed bacteria (*E.coli*, *S.aureus*, *S.epidermidis*) was markedly lower in ELBW infants when compared to term newborns and adults (Figure 3B). Consistent with results obtained with stimulation with bacterial agonists, leukocytes from term newborns showed a stronger response than adults to heat-killed *E.coli* and *S.epidermidis* (Figure 3B).

### Markers of innate immunity correlate with birth weight

Data from 60 patients were available to correlate birth weight and the expression of receptors of the innate immunity. A significant correlation was found between birth weight and surface receptor levels on phagocytes. Birth weight positively correlated with surface neutrophil expression of MD-2 (rho 0.62, P*<0.001*), CD14 (rho 0.34, *P = 0.009*), and TLR2 (rho 0.34, *P = 0.009*), but not TLR4 (rho 0.048, *P = 0.718*). Birth weight correlated with monocyte surface expression of MD-2 (rho 0.70, *P<0.001*), TLR4 (rho 0.33, *P = 0.011*), CD14 (rho 0.58, *P<0.001*), and TLR2 (rho 0.61, *P<0.001*). No correlation was found between birth weight and CD16, HLA-DR, and white blood cell count. Furthermore, birth weight correlated with levels of plasma-dependent opsonophagocytis for *E.coli* (rho 0.52, *P<0.001*), *S.aureus* (rho 0.55, *P = 0.001*), and *S.epidermidis* (rho 0.54, *P<0.001*). Finally, birth weight also positively correlated with levels of inflammatory cytokines in conditioned plasma from whole blood stimulated with LPS (rho 0.66, *P<0.001*), PAM_3_CSK_4_ (rho 0.57, *P<0.001*), heat-killed *E.coli* (rho 0.59, *P<0.001*), *S.aureus* (rho 0.54, *P<0.001*), and *S. epidermidis* (rho 0.56, *P<0.001*).

### Treatment of whole blood from premature infants with interferon-γ reverses the defect in anti-bacterial innate immunity

**Figure 4:**
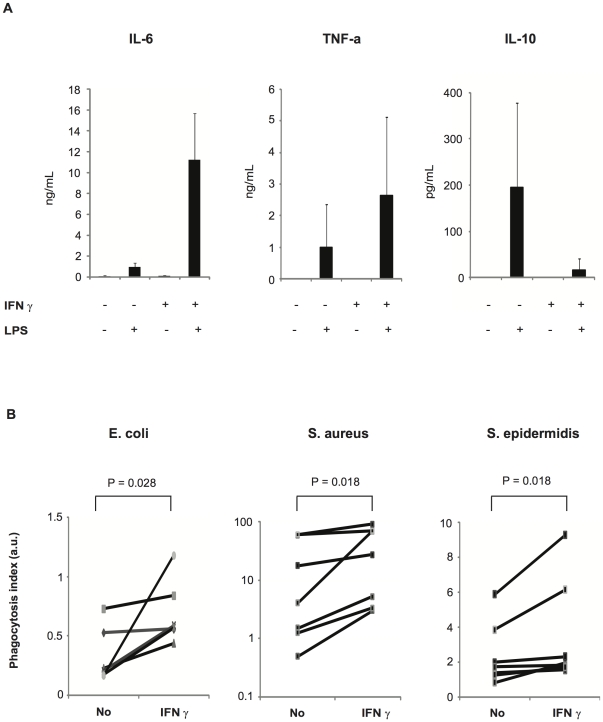
LPS responsiveness of leukocytes and opsonic capacity of plasma from premature infants with and without the addition of interferon-γ. (A) Whole blood from 7 extremely premature infants (born before 28 wks of gestational age) was treated *ex vivo* or not for 12 hrs with interferon (IFN)-γ. LPS was then added for an additional 12 hrs and IL-6, TNF-α, and IL-10 were measured (mean ± SEM) in conditioned plasma. (B) The plasma from whole blood obtained in seven extremely premature infants (born before 28 wks of gestational age) treated *ex vivo* or not with for 12 hrs with IFN-γ was incubated with fluorescent *E.coli*, *S.aureus* and *S.epidermidis* (opsonisation). Opsonised bacteria were then incubated with neutrophil-like HL-60 cells for 40 min; intracellular fluorescence was measured by flow cytometry, and expressed as mean phagocytic index (± SEM). Statistical significance was tested with Wilcoxon matched-pairs signed rank test.

Since we observed a marked defect in innate immune receptors and activity against bacteria in circulating leukocytes from premature infants, we tested the hypothesis that the immunomodulating cytokine interferon-γ may reverse this phenotype [13]. A 12 hr-course *ex vivo* treatment of whole blood with IFN-γ restored LPS responsiveness of circulating leukocytes from premature infants to levels measured in control adults (11.2±4.5 ng/mL IL-6 in conditioned supernatants from IFN-γ treated neonate leukocytes stimulated with LPS vs. 16.7±2.8 in untreated leukocytes from healthy adults stimulated with LPS) (Figure 4A). This was observed for the two pro-inflammatory cytokines TNF-α and IL-6. In contrast, the anti-inflammatory and immuno-suppressive IL-10 cytokine was decreased after IFN-γ treatment when whole blood was stimulated with LPS. Furthermore, phagocytosis of *E.coli* and staphylococci by DMSO-differentiated HL-60 cells was increased significantly when plasma was obtained in premature whole blood treated *ex vivo* by IFN-γ (Figure 4B). Altogether these results suggest that IFN-γ matured circulating leukocytes by increasing the expression of receptors of the innate immunity and opsonins, and polarized the immune response towards a Th1 response [14].

## Discussion

Herein, we show that premature infants, and particularly neonates with extremely low birth weight (ELBW), are born with an immature innate immunity resulting in a profound defect in the recognition and the clearance of bacteria. These defects in the capacity to mount a proper inflammatory response and to phagocytise bacteria are likely to account for the high incidence of neonatal sepsis associated with extreme prematurity. Importantly, a short treatment course with the immunomodulating cytokine IFN-γ was able to enhance proinflammatory and phagocytic responses in leukocytes from premature infants.

A low leukocyte TLRs expression in premature infants has been suggested in other studies [9], [10], [11]. We show in the present work that key receptors for the recognition of bacteria are remarkably low in neutrophils and monocytes from extremely premature newborns. The marked defective expression of MD-2, a key component of the LPS receptor, may well be responsible for the lack of innate inflammatory and immune response to bacteria observed in premature infants [15], and hyporesponsiveness of monocytes to LPS from premature infants [9]. Given the essential role of MD-2 in the control of Gram-negative infections, it is a possibility that the observed MD-2 deficiency accounts for the propensity of premature infants to developed life-threatening *E.coli* sepsis and meningitis [16], [17]. Furthermore, a recent study suggests that the absence of MD-2 may participate in the pathogenesis of necrotizing enterocolitis in premature infants [18].

Phagocytosis was also severely impaired in premature infants. Plasma-dependent opsonophagocytosis of *S.aureus* was found to be low both in premature and term newborns and was only efficient in control adults. This suggests that plasma opsonins important for *S.aureus* phagocytosis appear in the postnatal period. Plasma opsonophagocytosis of *S.epidermidis* was low in ELBW infants but not in older infants. This is in accordance with epidemiological data showing that extreme prematurity is associated with an elevated incidence of *S.epidermidis* neonatal sepsis [16]. An initial pro-inflammatory reaction is key to control bacterial infection [19]. A marked decrease in the inflammatory response to bacteria and bacterial agonists was principally observed in leukocytes from ELBW infants. This may be due to the low levels of receptors for bacterial products displayed at the surface of phagocytes from premature infants. In contrast, term newborns displayed a robust pro-inflammatory response, even greater than that of adult controls. This phenomenon was also observed by others and may be related to the lack of a proper counter-regulation by the anti-inflammatory cytokine IL-10, and/or to a leukocyte priming at the time of delivery [20], [21], [22], [23]. Interestingly, the LPS hyperresponsiveness observed in term newborn leukocytes is repressed in early post-natal months, and progressively re-increase to an adult response level within the first year of life [24]. The vast majority of mothers who delivered premature infants were treated with antenatal glucocorticoids in order to promote lung maturation; only 3 ELBW infants were born from untreated mothers due to emergency delivery. Both the inflammatory response and opsonophagocytosis were similar between ELBW infants from mothers treated or not, suggesting that antenatal steroid therapy did not influence postnatal innate immune response (data not shown).

Altogether, the extreme susceptibility of preterm newborns to invasive bacterial infection may be explained by the observed defects in both opsonophagocytosis and pro-inflammatory responses in this patient population. We have focused our work only on selective aspects of innate immune responses, i.e. inflammation and phagocytosis. Neutrophil chemotaxis was for example not studied but is evidently important in the context of innate immune defenses to pathogens, and has been shown to be defective in newborn and premature infants [25]. This defective migration capacity of neutrophils may also account for an elevated rate of neonatal sepsis. Interestingly, C-X-C chemokine receptor 2 (CXCR2) surface expression was recently shown to be closely related to TLRs mediated inflammatory response through the modulation of phosphatidylinositol 3-kinase (PI3K) [26], [27]. On the other hand, intracellular signaling proteins in the TLR pathway, such as MyD88 and IRF5, were also found at low levels in premature infant leukocytes [11]. The concomitant defective LPS responsiveness and chemotaxis of blood leukocytes from extremely low birth weight infants raises the question of a possible defective cross talk between TLRs and chemotaxis *via* the PI3K pathway.

It seems likely that extremely premature newborns are born with an immature innate immune system, possibly because of the lack of physiological immune maturation happening late in gestation, close to the term. In our study, we showed that a short *ex vivo* treatment with Th1-inducing cytokine interferon-γ matured leukocytes from preterm infants. This situation is reminiscent of that observed in critically ill patients who suffer from immunoparalysis or leukocyte reprogramming [19], [28], [29]. Phagocytes from septic patients lose their ability to mount a proper pro-inflammatory response to bacterial pathogens and this phenomenon is believed to play a significant role in the development of secondary bacterial infections [25]. Boosting the immune system with IFN-γ or GM-CSF has become a rational strategy and was associated with favourable outcomes in patients with sepsis [13], [30], [31]. Herein, we showed that *ex vivo* stimulation of whole blood from very premature infants with IFN-γ increased both pro-inflammatory response to LPS and plasma opsonophagocytosis capacity.

We tested the opsonophagocytic activity of conditioned plasma by incubating bacteria with conditioned supernatants from IFN-γ-treated leukocytes [32]. We found an increase in bacterial phagocytosis. This is most likely in relation with opsonins adsorbed by bacteria, opsonins that were produced by neonate leukocytes in response to the IFN-γ treatment. Among the possible IFN-g inducible opsonins, we favor pentraxins (C-reactive protein, PTX3), and soluble MD-2 [33], although this was not tested. Since opsonised bacteria were extensively washed before given to HL-60 cells for phagocytosis, it is unlikely that unbound soluble factors from conditioned supernatants were transferred together with bacteria and have influenced HL-60 phagocytic capacity.

Premature infants are known to have a “Th2” dominance, with a decreased IFN-γ production by NK cells, partly related to epigenetic regulation of the *IFN-γ* gene expression, but also due to a decreased IL-12 and IL-18 production by antigen-presenting cells [14], [34]. Skewing the immune response toward a Th2 response appears to be an evolutionary adaptation protecting placenta and foetus against maternal immune-mediated rejection [35]. In the foetus, Th2 polarization is orchestrated by different regulatory proteins, such as IL-10, prostaglandin E2, and progesterone [36], [37]. Effects of IFN-γ on the immune system are numerous, including the upregulation of TLRs expression, and particularly TLR4 and MD-2, as well as in the stimulation of phagocytosis [24], [33], [38]. Interestingly, the *ex-vivo* IFN-γ treatment of newborn whole blood increased leukocyte chemotaxis, as well as bacterial and fungal killing [39], [40], [41]. IFN-γ therapy was also shown to reduce the occurrence of severe infections in children with a chronic granulomatous disease [42]. We propose that our results serve as a proof-of-concept of pharmacologically-induced maturation of innate immunity in premature infants, similarly to foetal lung maturation by antenatal treatment with corticosteroids [43]. Although IFN-γ therapy was not associated with significant toxicity in children with chronic granulomatous disease [42], this cytokine can induce fever and flu-like symptoms, which may mask signs of sepsis in the fragile population of premature infants. In addition, IFN-γ could, at least theoretically, impair glial differentiation, and participate in the development of periventricular leukomalacia [44], [45]. Another phagocyte maturating agent, GM-CSF, was shown to reverse *ex-vivo* sepsis-associated monocytic immunosuppression in adults and could be an alternative to IFN-γ [30]. GM-CSF was administered in a controlled trial in critically ill premature infants patients performed and failed to demonstrate protection against neonatal sepsis and did not significantly modify the outcome [46]. The reason of the relative inefficiency of GM-CSF in premature infants has been illustrated by the selective impaired anti-apoptotic effect (alteration of cytochrome C release and caspase-3 and -9 activity) of GM-CSF on newborn neutrophils [47]. Furthermore, the absence of a prophylactic effect of GM-CSF may have been explained by the already increased plasma levels measured in premature infants [48].

In conclusion, we show in the present study that key innate immune functions of phagocytes are severely impaired in premature infants, particularly in those with extremely low birth weight. We demonstrate that the innate immune system matures with gestational age. Although a definite relationship between this innate immune defect and the high incidence of neonatal sepsis in the population of premature neonates has not been formally established, it is tempting to relate the two phenomena. We also show that the innate immune deficiency in extreme premature infants can be reversed by treatment with IFN-γ, opening a new therapeutic avenue to prevent the development of bacterial infections in premature newborns.

## Materials and Methods

### Patients and Samples

Ninety patients were included in the study. No patients had sepsis or suspected chorioamionitis at the time of delivery. Extremely low birth weight (ELBW, born before the 28^th^ week of gestational age) premature infants were compared to very low birth weight (VLBW, born between 28 and 32 wks of gestational age) premature infants, term newborns (TN) and control adults (CA). The study was approved by the University Hospital of Geneva Ethics Committee and registered to ClinicalTrials.gov (identifier: NCT00866567), and a written informed consent was obtained from parents. Between March and June 2009, all consecutive premature infants delivered were included. Heparinised blood obtained from umbilical cord was collected immediately after delivery and processed in the laboratory within 12 hrs after collection. Plasma was obtained by centrifugation of heparinised whole blood and stored at −70°C. A complete blood cell count was performed in all premature infants, and in a subset of term newborns. Control blood from adults was obtained by venipuncture and treated as newborn's blood.

### Cells and Reagents

Human HL-60 cell line was obtained from American Type Culture Collection (ATCC, Manassas, VA) and cultured in RPMI supplemented with 10% FCS (Amimed, Bioconcept, Basel, Switzerland), 2 mM L-glutamine, 10 mM HEPES, 50 µg/mL gentamicine and 50 U/mL penicillin. HL-60 cells were differentiated into neutrophil-like cells with 1.2% dimethylsulfoxyde (DMSO, Sigma, St Louis, MO) for 11 days [49]. The expression of Fc-γ receptors CD16, CD32, and CD64 in differentiated HL-60 cells was determined by flow cytometry using appropriate antibodies (BD Biosciences Pharmigen, San Diego, CA, Figure S1). Human embryonic kidney (HEK) 293 cells expressing human TLR4 (TLR4-HEK293) were cultured in DMEM supplemented with 10% FCS, 2 mM L-glutamine, 10 mM HEPES, 50 µg/mL gentamicine and 50 U/mL penicillin [50]. *S.epidermidis*, *S.aureus*, and *E.coli* were obtained from the Laboratory of Bacteriology of our institution and originated from positive blood cultures from premature infants with neonatal sepsis. Heat-killed *S.epidermidis* were labeled with fluorescein isothiocyanate (FITC) by using NHS-Fluorescein (Pierce, Rockford, IL) according to the manufacturer's protocol and stored at −20°C.

### Surface Receptors Labeling and Soluble MD-2 Activity

Leukocytes from heparinised blood were isolated from red blood cells using a gelatin gradient [6]. Remaining red blood cells were lysed with a 0.15 M ammonium chloride shock. Leukocytes were resuspended in PBS containing 3% heat inactivated human plasma (stock from a single donor). To measure receptor surface expression, leukocytes were incubated with the following murine antibodies: anti-huCD14 28C5 MAb (a gift from P.S. Tobias, The Scripps Research Institute, La Jolla, USA), anti-huTLR2 T2.5 MAb (eBioscience, San Diego, CA), anti-huTLR4 15C1 MAb, anti-huMD-2 18H10 MAb (gifts from G. Elson, NovImmune SA, Geneva, Switzerland) and a secondary APC-labeled anti-mouse antibody (Molecular Probes, Leiden, The Netherlands) as described [6], [50], [51], [52]. Polymorphonuclear neutrophil (PMN), monocyte, and lymphocyte populations were gated according to their forward and side-scatter characteristics, their CD16 and HLA-DR expression, and analyzed by flow cytometry. The presence of soluble MD-2 activity in plasma was assessed using an assay previously described [50], [51]. In brief, TLR4-HEK293 cells were stimulated with 30 ng/mL ultrapure *E.coli* LCD25 LPS (Invivogen) in the presence of patient's plasma for 21 hours or human recombinant soluble MD-2 (1 µg/mL) as a positive control, as described [50], [51]. The secretion of IL-8 in conditioned supernatants was quantified by ELISA [50], [51].

### Measurement of Opsonophagocytosis

The capacity of patient's plasma to enhance phagocytosis of bacteria was performed using neutrophil-like HL-60 cells differentiated for 11 days with DMSO. In a subset of patients from each group, patients' neutrophils were used as phagocytes. Fluorescent *E. coli* and *S. aureus* (6×10^9^ bacteria/mL; BODIPY FL bioparticles; Invitrogen), and fluorescent *S. epidermidis* (2.5×10^9^ bacteria/mL) were incubated for 1 hr at 37°C in PBS containing 5% decomplemented patient's plasma (opsonization period). After washing, plasma-opsonised fluorescent bacteria were incubated with differentiated HL-60 or patient's neutrophils at a bacteria-to-cell ratio of 50∶1 for *E coli*, 2∶1 for *S.aureus*, and 20∶1 for *S.epidermidis* for 20 to 40 minutes (10 to 20 minutes with primary neutrophils) in the same buffer as that used for the opsonisation period. Cells were then put on ice, washed once and resuspended in Hank's buffered salt solution. To only quantify internalized bacteria, extracellular fluorescence was quenched with 0.05% Trypan blue (Invitrogen). Phagocytosis inhibition by cytochalasin D (Sigma, 30 minutes treatment before adding fluorescent bacteria) served as a negative control [51]. The amount of phagocytosis was quantified by the calculation of the phagocytic index (PI). PI was defined as the geometric mean of fluorescence of gated cells (with predefined M1 gate corresponding to 99% fluorescence of cytochalasin D negative control) multiplied by the percentage of positive cells [53].

### Whole Blood Stimulation Assay

Fresh heparinised blood from patients was diluted 1∶2 with RPMI and incubated with ultrapure *E. coli* K12LCD25 LPS (Invivogen, San Diego, CA; 2.5 ng/mL), the lipopeptide PAM_3_CSK_4_ (Invivogen; 25 ng/mL) or with heat-killed bacteria Blood was stimulated with various concentrations of bacteria, ranging from 10^4^ to 10^8^ bacteria/mL, and results were expressed with cytokine levels obtained with a fixed concentration of 3.7×10^6^ bacteria/mL for *S.epidermidis* and *S. aureus*, and 1.56×10^6^ for *E.coli*. IL-6 and IL-8 levels were measured in conditioned supernatants by ELISA as described [6]. For each experiment with patient's blood, blood from an adult control was treated in parallel (positive control).

### *Ex-vivo* Treatment with Interferon-γ

In some patients, whole blood was diluted 1∶2 with RPMI and was cultured with 50 ng/mL interferon-γ (IFN-γ; Imukin®, Boehringer Ingelheim, Germany) for 12 hrs at 37°C. Diluted blood was then stimulated with ultrapure *E.coli* K12LCD25 LPS (10 ng/mL) for another 12 hrs. IL-6 levels were measured in conditioned supernatants by ELISA, whereas TNF-α, and IL-10 were measured with the BD cytometric bead array system (BD Biosciences). Opsonophagocytosis activity of IFN-γ-conditioned plasma was measured using differentiated HL-60 incubated with fluorescent *E.coli*, *S.aureus* and *S.epidermidis*, as described above. Bacteria were incubated with conditioned plasma, and then extensively washed before being put together with neutrophils for the phagocytosis assay.

### Statistics

We calculated that ≥20 patients per groups were needed to identify a 33% variation in measured variables with a power of >80%, and an α error <5%. Continuous data were expressed as median plus interquartile range or mean ± standard error of the mean (SEM) when appropriate. Differences between >2 groups were tested using the Kruskal-Wallis test before performing pairwise comparisons using the Mann-Whitney *U* test. [54] Correlations were determined using the Spearman rank correlation test, and Wilcoxon matched-pairs signed rank test was applied to analyze continuous series.

## Supporting Information

Figure S1 **Expression of surface receptors by neutrophil-like DMSO-differentiated HL-60 cells.** The surface expression of Toll-like receptor (TLR4), TLR2, CD14, MD-2, and Fcγ receptors CD16, CD32, CD64 was measured by flow cytometry using appropriate monoclonal antibodies in undifferentiated HL-60 cells (light grey), and in HL-60 cells differentiated with DMSO for 11 days (black), and compared to an isotype control antibody (dark grey).
(TIFF)

Figure S2 **Opsonic capacity of autologous plasma using autologous neutrophils as phagocytes.** Phagocytosis of fluorescent *E.coli*, *S.aureus*, and *S.epidermidis* (opsonised with autologous plasma) by neutrophils from ELBW, extremely low birth weight premature infants born before 28 wks of gestational age, N = 8; VLBW, very low birth weight premature infants born between 28–32 wks of gestational age, N = 6; TN, term newborns, N = 8; and CA, control adults, N = 13). Phagocytosis was measured by flow cytometry after 20 min and expressed as mean phagocytic index ± SEM).
(TIFF)
