# Supplementary figures and images for: Innate Immune Deficiency of Extremely Premature Neonates Can Be Reversed by Interferon-γ
Source: PLoS One. 2012 Mar 12;7(3):e32863. doi: 10.1371/journal.pone.0032863 (PMC3299693; doi:10.1371/journal.pone.0032863)

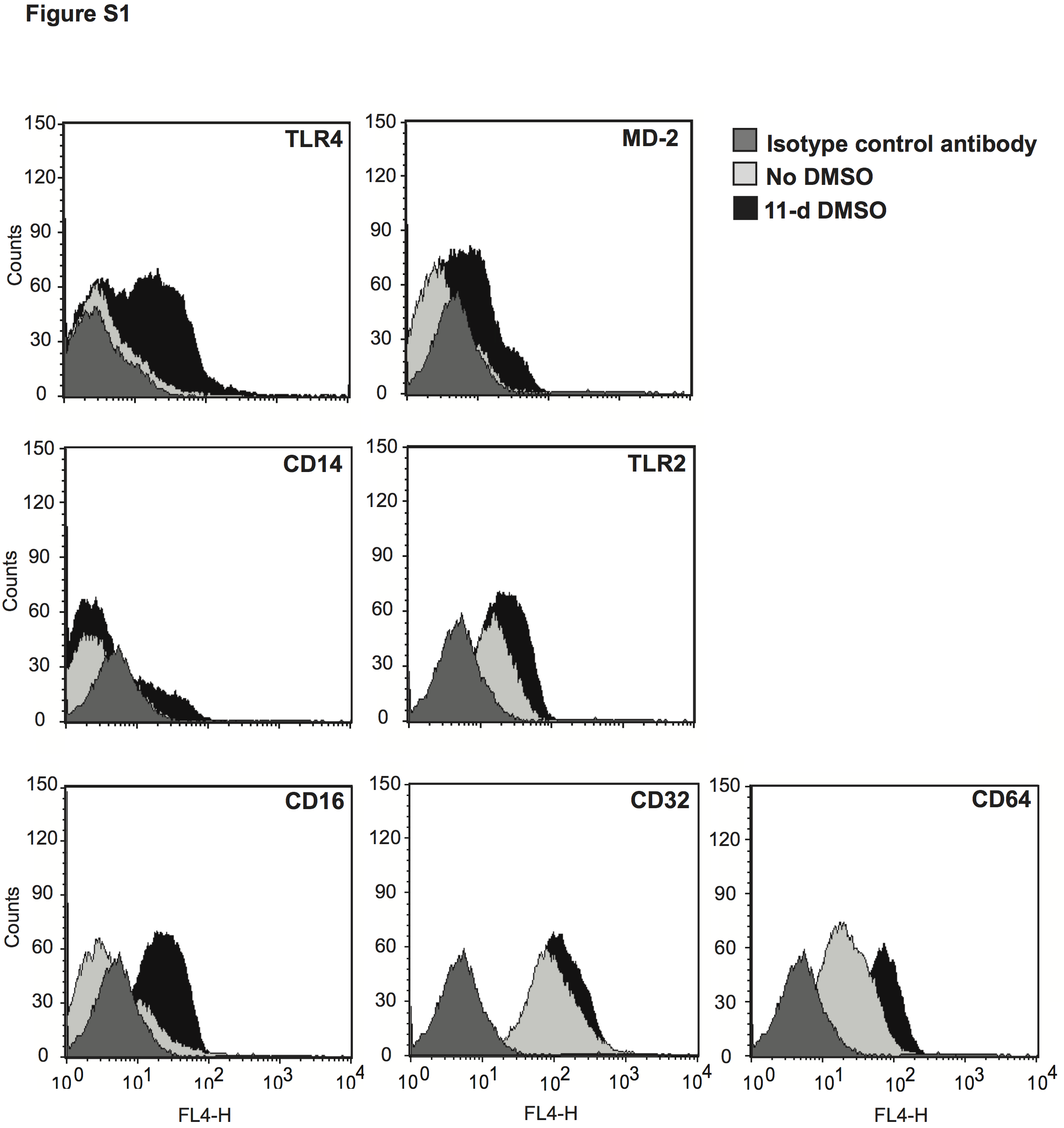

Supplement: Figure S1 — Expression of surface receptors by neutrophil-like DMSO-differentiated HL-60 cells. The surface expression of Toll-like receptor (TLR4), TLR2, CD14, MD-2, and Fcγ receptors CD16, CD32, CD64 was measured by flow cytometry using appropriate monoclonal antibodies in undifferentiated HL-60 cells (light grey), and in HL-60 cells differentiated with DMSO for 11 days (black), and compared to an isotype control antibody (dark grey). (TIFF) [file pone.0032863.s001.tiff]

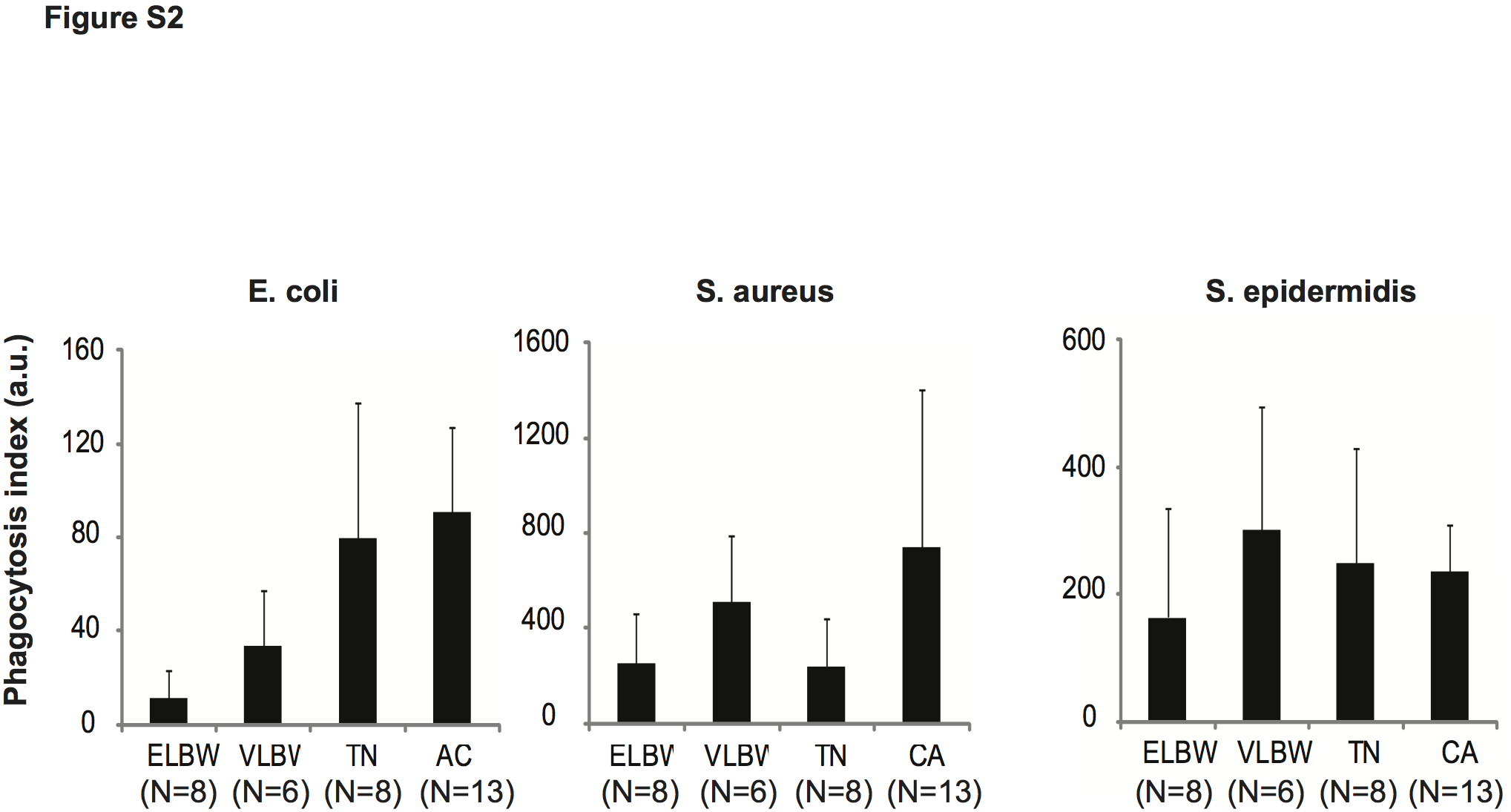

Supplement: Figure S2 — Opsonic capacity of autologous plasma using autologous neutrophils as phagocytes. Phagocytosis of fluorescent E.coli, S.aureus, and S.epidermidis (opsonised with autologous plasma) by neutrophils from ELBW, extremely low birth weight premature infants born before 28 wks of gestational age, N = 8; VLBW, very low birth weight premature infants born between 28–32 wks of gestational age, N = 6; TN, term newborns, N = 8; and CA, control adults, N = 13). Phagocytosis was measured by flow cytometry after 20 min and expressed as mean phagocytic index ± SEM). (TIFF) [file pone.0032863.s002.tiff]
